# Babesia divergens egress from host cells is orchestrated by essential and druggable kinases and proteases

**DOI:** 10.21203/rs.3.rs-2553721/v1

**Published:** 2023-02-28

**Authors:** Brendan Elsworth, Caroline Keroack, Yasaman Rezvani, Aditya Paul, Keare Barazorda, Jacob Tennessen, Samantha Sack, Cristina Moreira, Marc-Jan Gubbels, Marvin Meyers, Kourosh Zarringhalam, Manoj Duraisingh

**Affiliations:** Harvard T.H. Chan School of Public Health; Harvard T.H. Chan School of Public Health; University of Massachusetts Boston; Harvard T.H. Chan School of Public Health; Harvard T.H. Chan School of Public Health; Harvard T.H. Chan School of Public Health; Harvard T.H. Chan School of Public Health; Harvard T.H. Chan School of Public Health; Boston College; Saint Louis University; University of Massachusetts Boston; Harvard T.H. Chan School of Public Health

## Abstract

Apicomplexan egress from host cells is fundamental to the spread of infection and is poorly characterized in *Babesia* spp., parasites of veterinary importance and emerging zoonoses. Through the use of video microscopy, transcriptomics and chemical genetics, we have implicated signaling, proteases and gliding motility as key drivers of egress by *Babesia divergens*. We developed reverse genetics to perform a knockdown screen of putative mediators of egress, identifying kinases and proteases involved in distinct steps of egress (ASP3, PKG and CDPK4) and invasion (ASP2, ASP3 and PKG). Inhibition of egress leads to continued intracellular replication, indicating exit from the replication cycle is uncoupled from egress. Chemical genetics validated PKG, ASP2 and ASP3 as druggable targets in *Babesia* spp. All taken together, egress in *B. divergens* more closely resembles *T. gondii* than the more evolutionarily-related *Plasmodium* spp. We have established a molecular framework for biological and translational studies of *B. divergens* egress.

## Introduction

*Babesia* spp. are tick-borne pathogens that infect, grow in, and eventually destroy the red blood cells (RBCs) of their vertebrate host. Several *Babesia* spp., including *B. microti, B. divergens* and *B. duncani*, are emerging zoonotic pathogens that can cause severe and even fatal disease^1^. *Babesia* spp. also cause significant economic losses globally due to disease in livestock and companion animals, especially *B. divergens, B. bovis* and *B. bigemina* in cattle^2^. Current treatment and prevention options for human and veterinary babesiosis are limited and suffer from poor efficacy, spontaneous resistance, severe side effects or render the animal products unsuitable for human consumption^1,3^.

The *Babesia* genus is within the Apicomplexa phylum, which consists of single-celled obligate intracellular parasites. Apicomplexa include the human and veterinary pathogens, *Babesia* spp., *Plasmodium* spp., *Toxoplasma* spp., *Cryptosporidium* spp., *Theileria* spp. and *Eimeria* spp., amongst others. *Babesia* spp. are most closely related to *Theileria* spp. and *Plasmodium* spp., which all share the same vertebrate RBC niche for part of their lifecycle. The vast majority of apicomplexan cellular and molecular biology research has been performed in *Plasmodium* spp. and *Toxoplasma gondii*. Knowledge of a wider range of apicomplexan biology will hone in on conserved and essential functions that could be key targets to develop broad spectrum anti-apicomplexan drugs. *Babesia* spp. represent a minimalized apicomplexan genome with *B. divergens* having 4187 genes in comparison to *P. falciparum* (5720 genes) or *T. gondii* (8920 genes) (VEuPathDB v61). There are many similarities between *Plasmodium* spp., *Toxoplasma* spp. and *Babesia* spp., such as the presence of an apicoplast and apical invasion organelles. However, there are notable differences, such as *Babesia* spp. degrading their parasitophorous vacuole immediately after invasion and replicating in the host cell cytoplasm. The mechanisms of cell division also vary between apicomplexan parasites (reviewed in Gubbels, et al.^4^). In this study, a replication cycle refers to one cell undergoing division to form two (*Babesia* spp. and *T. gondii*) or more (*Plasmodium* spp.) daughter cells. A lytic cycle refers to the time from when a host cell is first invaded to when parasites egress from the same host cell, which may include multiple replication cycles in the case of *Babesia* spp. and *T. gondii*, but only one for *Plasmodium* spp. (Figure S1A)^5^.

Parasite egress from the host cells remains poorly characterized in *Babesia* spp. Egress in the two previously studied apicomplexans, *Plasmodium* spp. and *T. gondii* (reviewed in^6,7^), begins when either intrinsic or extrinsic signals activate signaling pathways in the parasite that ultimately result in the release of parasite secretory organelles, the micronemes/exonemes, into the host cell cytoplasm. The contents of these organelles include proteases, phospholipases and perforin-like proteins (PLPs), which help permeabilize and eventually rupture the parasitophorous vacuole membrane (PVM) and host cell membrane, allowing the parasite to egress from the host cell. Many of the molecular players in the egress program are shared between *Plasmodium* spp. and *T. gondii*, including cGMP-dependent kinase (PKG), calcium-dependent protein kinases (CDPKs), aspartyl proteases, lipid and calcium signaling^8,9^. However, there are key differences, such as protein kinase A (PKA) repressing egress in *T. gondii* but is instead required for invasion in *P. falciparum*^10-12^. Significant effort has been utilized to develop inhibitors of egress in *Plasmodium* spp. and *T. gondii*, with the potential to develop pan-apicomplexan drugs (reviewed in^13,14^).

Here, we have employed a range of cellular, genetic and genomic approaches to produce a foundational framework defining the key cellular features of *B. divergens* egress and identify the molecular mediators of these processes through functional analysis. We have developed stable transfection, a CRISPR/Cas9 system and inducible knockdown systems for *B. divergens*. These genetic systems were used in combination with small molecule inhibitors, to determine that the *B. divergens* kinases, PKG and CDPK4, and the aspartyl proteases, ASP2 and ASP3, are required for separate sequential steps in egress and invasion of the host RBC. Strikingly, inhibition of egress resulted in continued intracellular replication within a single RBC, suggesting the default pathway in these parasites is to continue replicating in the absence of either an egress signal or the downstream host lytic effector molecules. Moreover, chemical genetic approaches demonstrate that these conserved essential molecules present validated druggable targets in *Babesia* spp.

## Results

### An induced egress assay to study B. divergens host cell egress and invasion

**Figure 1:**
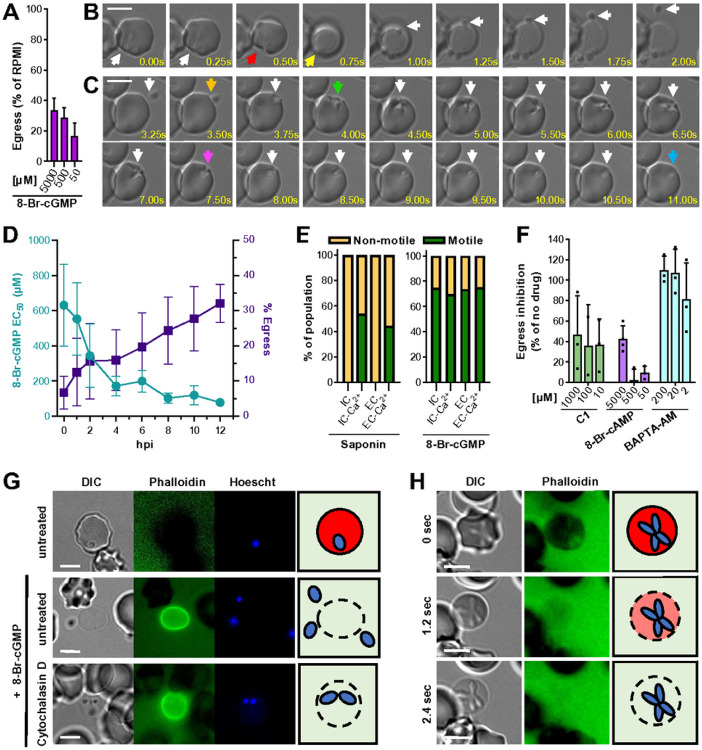
Dynamics of host cell egress and invasion by *B. divergens*. **(A)** Percentage of parasites that egress when treated with 8-Br-cGMP. Data is normalized to an RPMI treated control (0%) **(B and C)** Time-lapse microscopy of *B. divergens* egress (B) and invasion (C). A and B follow the same parasite egressing from one cell and invading a new RBC. Arrows indicate as follows: Red - initial deformation of the RBC in egress. Yellow - ‘rounding-up’ associated with permeabilization of the RBC. Orange - initial RBC contact in invasion. Green - initial deformation of the RBC. Purple – beginning of internalization. Blue – completion of invasion. White – follows the parasite. **(D)** Egress was induced throughout one replication cycle using a range of 8-Br-cGMP concentrations. The EC_50_ of 8-Br-cGMP is shown in teal and the percentage of parasites that egress at 2 mM 8-Br-cGMP is shown in purple. Data is normalized to 0% being a no 8-Br-cGMP treated control. **(E)** Percentage of parasites that become motile when the iRBC is permeabilized by saponin, or induced to egress with 8-Br-cGMP, in the stated buffer. IC and EC refer to intracellular and extracellular buffer with or without 2 mM calcium (Ca^2+^). Data is pooled from multiple experiments. >30 iRBCs were used for each condition. **(F)** Screen of small molecules for inhibition of 8-Br-cGMP mediated induced egress. Data is normalized to 100% as a no 8-Br-cGMP control and 0% being RPMI plus 8-Br-cGMP. **(G)** *B. divergens* iRBCs pretreated with cytochalasin D and induced to egress with 500 μM 8-Br-cGMP. Phalloidin stains the permeabilized RBC. **(H)** Time-lapse images showing RBC permeabilization by cytochalasin D-treated *B. divergens* during induced egress. Phalloidin is excluded from the intact RBC. For A, F and H, the mean ± SD of three biological experiments performed in technical triplicate is shown. The scale bar in B-D is 5 μm.

To make the study of egress accessible in *B. divergens*, we developed a method to induce egress, which otherwise occurs asynchronously in *in vitro* culture. A flow cytometry-based assay was used to screen for egress induction in *B. divergens* using known *T. gondii* egress-inducing compounds (Figs. 1A, S1B and S1D). 8-Br-cGMP, BIPPO (phosphodiesterase (PDE) inhibitor) and to a lesser extent, H89 (PKA inhibitor), induced egress and inhibited parasite replication (Figs. 1A, S1D-H)^12,15^. A summary of the putative *B. divergens* egress signaling pathway can be found in S1C.

To elucidate the cellular feature of *B. divergens* egress and invasion, we used video microscopy to follow 8-Br-cGMP mediated induced egress. As has been previously observed^16^, *B. divergens* egress is frequently initiated when the intracellular parasite contacts and deforms the host cell (observed in 76% (16/21) of egress events, Fig. 1B - red arrow). Soon after the initial deformation, the RBC ‘rounds up’ (mean ± S.D. = 2.6 s ± 3 s, n = 17) (Fig. 1B, yellow arrow). ‘Rounding’ is observed in *P. falciparum* immediately preceding PVM rupture and may be associated with increased PVM permeability, however, the mechanisms remains unclear^17-19^. In *B. divergens*, which lack a PVM, rounding is likely due to permeabilization of the RBC. *B. divergens* parasites then become motile and escape the permeabilized RBC to invade new RBCs (mean ± S.D. = 1 s ± 2.4 s, n = 28, to escape after host cell permeabilization). Upon contact with a new RBC, the parasite strongly deforms the host cell around itself (Fig. 1C, green to purple arrows; mean ± S.D. = 6 s ± 2.7 s, n = 14), a process known as pre-invasion in *P. falciparum*, followed by the internalization phase when the parasite enters the RBC with relatively little deformation of the RBC (Fig. 1C, purple to blue arrows, mean ± S.D. = 4.5 s ± 1.1 s, n = 10; Video S1)^20^. In contrast to *P. falciparum* egress that takes ~ 5–10 minutes (from initial PVM permeabilization to merozoite release)^18,21^, *B. divergens* egress is rapid (mean ± S.D. = 10.8 s ± 3.0 s, n = 10).

To test the ability of *B. divergens* to egress throughout replication, parasites were synchronized to a 20-minute window and egress was induced every 1–2 hrs. The increased sensitivity to 8-Br-cGMP and increased number of parasites that egress as the parasites mature suggest that while *B. divergens* can egress throughout the replication cycle, it is more strongly primed to egress when parasites are fully mature (Fig. 1D). This feature of *B. divergens* egress is similar to *T. gondii*, which can be induced to egress throughout the replication cycle, albeit at reduced efficiency during S and M/C phase^22^, whereas *P. falciparum* egress is restricted to a narrow window at the end of the lytic cycle^15,23^.

### Loss of host cell integrity induces B. divergens motility and egress

To test if *B. divergens* motility is induced by exposure to extracellular conditions, as has been demonstrated in *T. gondii*, we observed parasites after the RBC was lysed using a low concentration of saponin in buffers mimicking either intracellular (IC, 140 mM K^+^, 5 mM Na^+^) or extracellular (EC, 5 mM K^+^, 140 Na^+^) concentrations of potassium and sodium, plus or minus calcium (2 mM). Upon saponin-induced RBC lysis in EC-Ca^2+^ or IC-Ca^2+^ buffer, parasites became motile and were able to escape from the permeabilized RBC (Fig. 1E, Video S3). In contrast, parasites in either IC or EC buffer without Ca^2+^ did not become motile (Fig. 1E, Video S4). PKG activation, through the addition of 8-Br-cGMP, bypassed the requirement for extracellular calcium in either buffer to initiate gliding motility (Fig. 1E). Together these results suggest that *B. divergens* motility is induced by exposure to extracellular concentrations of Ca^2+^, likely acting upstream or in parallel of PKG, but does not respond to Na^+^ or K^+^ concentrations. Similarly, *T. gondii* can sense exposure to the extracellular environment through serum albumin, a drop in potassium and an increase in extracellular calcium, which induces microneme secretion and motility^22,24-28^. *P. falciparum* is also able to sense lipids and potassium which alter microneme secretion, egress or invasion, although their roles in the parasite are less clearly defined^29-31^. Extracellular calcium is required for efficient invasion, but not egress, of *P. falciparum*^30,32,33^.

## Chemical inhibition of PKG, proteases and calcium release all impair egress

To identify the molecular processes required for *B. divergens* egress, we used the flow cytometry-based egress assay to screen for compounds that inhibit 8-Br-cGMP-induced egress. The compounds were selected based on known egress inhibition in *P. falciparum* or *T. gondii*. BAPTA-AM, which chelates intracellular calcium, and Compound 1 (C1), an inhibitor of apicomplexan PKG, both inhibited 8-Br-cGMP induced egress (Fig. 1F). Inhibitors of serine proteases, including TPCK, TLCK and PMSF, reduced induced egress at similar concentrations to those that inhibit *P. falciparum* gametocyte egress, whereas inhibitors of other protease classes exhibit modest inhibition (< 20%) (Figure S1H)^34-36^. The short incubation time (15 min) may not identify inhibitors of certain proteases such as PfPMX/TgASP3 which act several hours prior to egress. Inhibitors of the lipid signaling pathway that inhibit egress in *T. gondii* and/or *P. falciparum*, including U73122 and propranolol which target PI-PLC and phosphatidic acid phosphatase, respectively, did not influence egress by *B. divergens*, whereas the diacylglycerol kinase inhibitor R59022 instead enhanced induced egress (Figure S1H)^27,29,37,38^. In line with our flow cytometry assay, E64d treated parasites showed no obvious defect in egress, motility or invasion by video microscopy (Figure S1J). Taken together, these data demonstrate a requirement for cGMP signaling, calcium signaling (both intracellular and extracellular), serine proteases and gliding motility for efficient egress by *B. divergens*. Unlike in *T. gondii*, release of calcium with the ionophore A23187 does not induce egress or motility in *B. divergens*, suggesting calcium release is required, but not sufficient for egress. We note that a previous study found that A23187 could induce egress in *B. bovis* which could be due to differences between species, host cells, or technical such as the extracellular concentration of calcium^39^.

### Motility is required for B. divergens merozoites to egress from permeabilized RBCs but not for permeabilization

The initial deformation of the RBC in egress appears to be caused by direct contact with the parasite indicating the parasites actinomyosin motor may be used to physically disrupt the host membrane (Fig. 1B)^16^. To test if motility is required during egress, egress was induced with 8-Br-cGMP in the presence of phalloidin-Alexa Fluor 488, which selectively stains the cytoskeletons of permeabilized RBCs, and cytochalasin D, which inhibits actin polymerization and gliding motility. Cytochalasin D-treated parasites can be induced to lyse the host cell, but do not escape the permeabilized cell (Fig. 1G-H, Video S2). Immediately preceding egress, the host cell becomes ruffled, but no localized deformation is observed, followed by the RBC rapidly showing a reduced diameter, becoming round and being infiltrated by phalloidin, demonstrating the RBC membrane has been permeabilized (Fig. 1H, Video S2). *T. gondii* secreted lytic factors (e.g. PLP1) and host calpains damage the host cell, allowing the motile parasite to escape the permeabilized cell^27,40,41^. The mechanisms of RBC lysis in asexual *Plasmodium* spp. remain unclear^42,43^. *B. bovis* PLP1 is not strictly required for egress, however, its absence does lead to continued intracellular replication (4 parasites per cell instead of 2 which is typical of *B. bovis*), suggestive of a partial egress defect^44^. In contrast to *P. falciparum* which fractures the RBC cytoskeleton during egress (Figure S1I)^45^, the permeabilized RBC remains relatively intact throughout egress in *B. divergens* (Figs. 1B-C, 1G-H)^16^. *P. falciparum* secretes proteases (SERA6) which degrade the RBC cytoskeleton, leading to the to rupture of the RBC to release free merozoites, thus bypassing the requirement for gliding motility in egress^17,46,47^. Consistent with this difference, *Babesia* spp. do not have an ortholog of any SERA protease nor an ortholog of the phospholipase TgLCAT/PbPL. Taken together, these data argue for a mechanism of host cell lysis in *B. divergens* relying centrally on secretion of lytic factors (e.g PLPs). While, we do not observe a requirement for parasite motility for host cell lysis, we cannot rule out that motility contributes towards cell lysis as has been observed in *T. gondii*^40^.

## Identification of putative egress, motility and invasion genes through transcriptomic analyses

**Figure 2:**
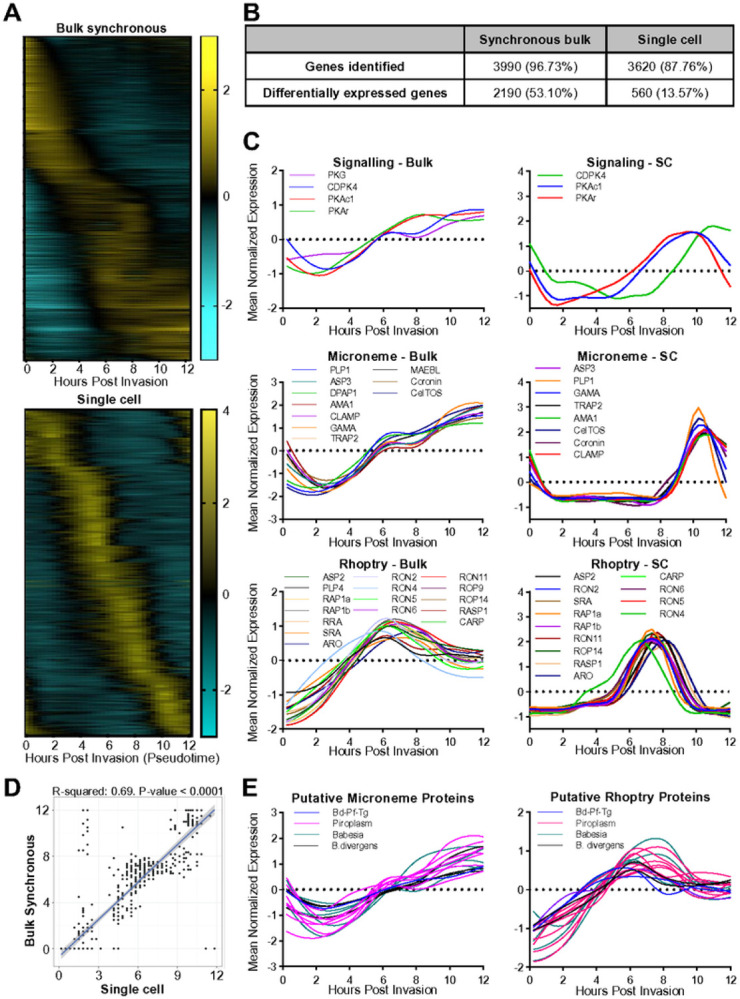
The *B. divergens* transcriptome reveals expression of putative egress and invasion genes. **(A)** Expression profile of all differentially expressed genes. **(B)** Number of genes identified by both transcriptomic approaches. Differentially expressed genes are defined as having a >1.5-fold change over time for the bulk transcriptome and a ≥2-fold-change, adj. p-value 0.001, for the single cell transcriptome. **(C)** The expression profiles from bulk synchronous and single-cell data of orthologs of known egress/invasion genes. **(D)** Correlation of timing of peak expression between both approaches. **(E)** The expression profiles from bulk synchronous data of putative novel egress/invasion genes. The single cell data was originally generated in 53.

The lytic cycle of *B. divergens* has been morphologically defined but remains poorly characterized at the molecular level in any *Babesia* spp.^5^ There are several *Babesia* spp. transcriptomes at different stages of the life cycle, however, there are no synchronous intraerythrocytic stage time course transcriptomes^48-52^. We generated a synchronous bulk transcriptome of one replication cycle (0–12 hrs) for *B. divergens*. As a parallel strategy, we utilized a recent asynchronous single-cell transcriptome where expression profiles of individual genes were generated using a pseudo-time analysis with the start of gene expression identified by cross-correlation analysis between the bulk and single-cell expression curves^53^. Genes that showed expression changes over time display a transcriptional cascade associated with “just-in-time” gene expression that has been observed in *Plasmodium* spp. and *T. gondii* (Figs. 2A and S2A)^54,55^. The number of genes detected and that display significant changes over time for each method can be found in Fig. 2B. The timing of peak expression between the bulk and single-cell transcriptomes was well correlated for the majority of genes, supporting the use of the pseudo-time data for downstream analysis (Fig. 2D).

93 *B. divergens* genes were identified as orthologs or family members of known egress, motility or invasion genes we have collated for apicomplexan parasites. 91 of these genes are expressed in *in vitro* culture and 67 showed expression changes over time (Table S1). Genes that are expected to co-localize within the same subcellular compartment (e.g. micronemes) based on orthology display similar expression profiles as has been observed in *Plasmodium* spp. and *T. gondii* (Figs. 2C and S2B)^54,56,57^. Secondary messenger-responsive proteins, including PKG, PKAc1, PKAr, and CDPK4 (PfCDPK4/TgCDPK3) peak ~ 8–10 hours post invasion (hpi) and remain high until 12 hpi when parasites naturally egress (Figs. 2C). Other putative members of the egress signaling pathway did not display stage-specific expression indicative of a role in egress or invasion (Table S1). The cysteine and aspartyl proteases, DPAP1 (no direct ortholog) and ASP3 (ortholog of PfPMX/TgASP3), respectively, display expression profiles matching that of microneme proteins (Figs. 2C). The aspartyl protease ASP2 (closely related to BdASP3) displays an expression profile matching rhoptry proteins (Figs. 2C). All nine *B. divergens* PLP genes are expressed (Data not shown). We found that only PLP1 (TgPLP1/PfPLP3) and PLP4 (no direct ortholog) display an expression profile matching other microneme and rhoptry proteins, respectively, suggestive of a role in egress and invasion or post-invasion PV breakdown, respectively (Figs. 2C).

Multiple invasion ligands were identified by orthology to known apicomplexan ligands and display expression profiles matching that of rhoptry or microneme proteins (Figs. 2E). These included TRAP2/P18, RAP1 and AMA1, which all have a demonstrated role in *Babesia* spp. invasion (Fig. 2C)^58-60^. The *B. divergens* orthologs of the *Plasmodium* spp. or *T. gondii* rhoptry protein, SRA, and microneme proteins, CLAMP, GAMA, MAEBL and CelTOS represent novel vaccine targets that have not been investigated in *Babesia* spp.^61-65^ Inner membrane complex (IMC) proteins, which are involved in cell structure and motility, are transcribed in three groups (Figure S2B), suggesting that IMC assembly with distinct profiles warrants future study.

A comparative transcriptomic approach using data from *P. falciparum* and *T. gondii* was used to identify novel proteins putatively involved in egress, motility or invasion (methods outlined in Figure S2C). 104 genes were identified, 31 of which were identified by multiple methods and whose orthologs have no known function (Figs. 2E; Table S1). Of the 8 genes found in all three species, 5 have a growth phenotype in the *T. gondii* CRISPR screen and the *P. falciparum piggyBac* screen (Table S1).

### A genetic screen of high priority candidates reveals the essentiality of PKG, CDPK4, ASP2 and ASP3 for parasite proliferation

We focused on 11 high priority candidates based on transcriptomics as well as orthology to apicomplexan egress genes, including the kinases, PKG, PKAc1, PKAc2, CDPK4, CDPK5 and CDPK7, the perforin-like proteins, PLP1 and PLP4, and the proteases, ASP2, ASP3 and DPAP1. PKG is conserved between *B. divergens, P. falciparum* and *T. gondii* and has a well-characterized role in egress and invasion in these latter species^66,67^. PKAc1 suppresses egress in *T. gondii* but is instead required for invasion by *P. falciparum*^10,12,68-71^. The CDPK family of proteins are also required for *P. falciparum* and *T. gondii* egress and invasion, although the direct orthologs (defined by reciprocal blast) do not always have the same function between species (Reviewed in^72,73^). PLPs are involved in host cell permeabilization and egress in *T. gondii, Plasmodium* spp. sexual stages and *B. bovis*^40,44,74-76^. PfDPAP family members and BdASP2/ASP3 orthologs (PfPMX, PfPMIX and TgASP3) are required for egress and/or invasion in *P. falciparum* or *T. gondii*^77-82^.

**Figure 3:**
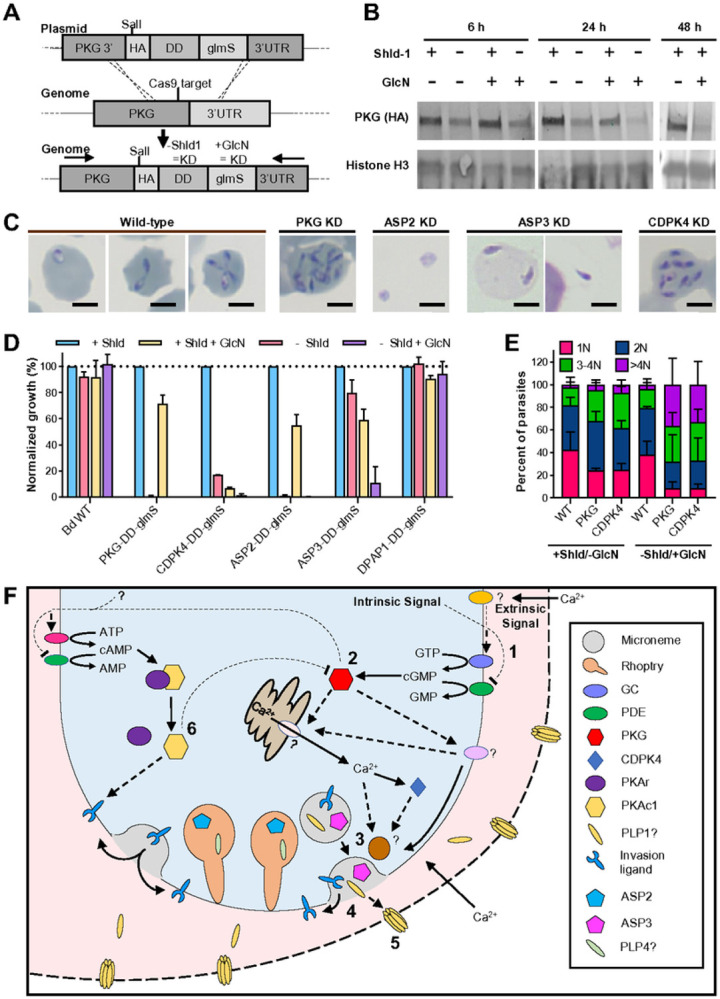
A system for inducible knockdown reveals an essential role for PKG, CDPK4, ASP2 and ASP3 in egress or invasion **(A)** CRISPR/Cas9 is used to introduce a HA-DD-glmS or HA-glmS tag to the 3’ end of *pkg* by homologous recombination. The same approach was used for all genes **(B)** Western blot analysis showing DD and glmS induced knockdown of PKG over time. **(C)** Growth of knockdown parasites over 3 days. Data are the mean ± SD of three biological experiments performed in technical triplicate. Data is normalized so +Shld is 100% **(D)** Giemsa stain showing phenotypes after 24-48 h of knockdown (KD). The scale bar is 3 μm. **(E)** The number of parasites per iRBC assessed by microscopy after knockdown (−Shld/+GlcN) of PKG or CDPK4 for 48 h. 100 cells were counted per condition. 3 biological replicates are shown **(F)** Model of the *Babesia* divergens lytic cycle. **1.** An extrinsic signal (calcium) or an intrinsic signal activates guanylate cyclase (GC) and/or inhibits a phosphodiesterase (PDE). **2.** The increased cGMP levels activate cGMP-dependent kinase (PKG), which leads to the release of calcium. **3.** Calcium, CDPK activity and potentially lipid signals lead to the fusion of micronemes to that parasite plasma membrane. **4.** The contents of the micronemes, which have been proteolytically mature by ASP3, are released into the RBC cytosol, including lytic factors (putatively PLP1), and invasion ligands onto the parasite surface **5.**Lytic factors act to permeabilize the RBC membrane. The permeabilized RBC membrane allows an influx of calcium, acting as a positive feedback loop. **6.** Unknown signals, potentially including PKG, lead to the activation of adenylate cyclase (AC) and/or inhibition of a PDE, which increases cAMP levels. The increased cAMP activates PKAc1, which is required for invasion. PKAc1 activity also reduces egress, potentially through inhibition of the PKG signaling pathway. Solid lines represent pathways with a high confidence based on available data for *B. divergens*. Dashed lines and proteins marked with a “?” indicate processes based primarily on the RNAseq data from this paper and orthology to *P. falciparum* or *T. gondii*.

To develop a stable transfection system for *B. divergens*, multiple transfection methods and selection drugs with established resistance markers were tested for stable transfection of a GFP reporter plasmid (Figure S3A and C) (summarized in Table S2). Nucleofection of isolated merozoites and blasticidin-S selection was the most efficient method and was used for all further transfections. A CRISPR/Cas9 system was generated to introduce a HA tag, as well as the *glmS* riboswitch with and without a destabilization domain (DD) inducible knockdown system in series to the 3’ end of each gene (Figs. 3A and S3B)^83,84^. Parasites containing the correct integration reached > 1% parasitemia 12–16 days after transfection for all constructs, except PKAc1, PLP1 and PLP4 which we were unable to tag (Figure S3F and H). PKG-HA-DD-glmS parasites were initially used to determine the effectiveness of the knockdown systems. Induction of the DD system, which induces protein degradation, generated a more rapid (~ 6 hrs) and stronger reduction of protein levels than the glmS system (Fig. 3B). The glmS system, which destabilizes mRNA, resulted in slower knockdown that was first observable by 24 hrs, but generated strong knockdown by 48 hrs (Fig. 3B). Combining both systems resulted in a stronger knockdown than either alone (Fig. 3B). This pattern was reflected in the effect of these knockdown systems on parasite growth when targeting PKG, CDPK4, ASP2 or ASP3, with the double knockdown producing the strongest defect in all lines, whereas the DD or glmS alone were not sufficient for all genes (Fig. 3D). DPAP1, PKAc2, CDPK5 and CDPK7 knockdown did not affect proliferation (Fig. 3D, data not shown).

## PKG and CDPK4 are essential for egress and their depletion results in continued intracellular replication

We analyzed knockdowns of PKG and CDPK4 following synchronized invasion to understand when the block in proliferation occurs. PKG or CDPK4 knockdown parasites continued to replicate within a single iRBC, to form up to 16 parasites per RBC by 40 hpi (Figs. 3C-E and S3I). The fraction of parasites that replicate to > 4 per iRBC is dependent on the degree of knockdown (Fig. 3E). Knockdown of PKG prevented induced egress by 8-Br-cGMP, further supporting that 8-Br-cGMP induces egress through activation of PKG (Figure S3D). The reduced susceptibility to 8-Br-cGMP mediated induced egress in PKG-HA-DD-glmS parasites without inducing knockdown compared to WT parasites is likely due to a partial knockdown in the absence of induction, due to tagging as observed in other parasite recombinants (Figure S3D)^85,86^. A model of the molecular pathways involved in *B. divergens* egress can be found in Fig. 3F.

## The proteases ASP2 and ASP3 are required for egress and invasion

**Figure 4:**
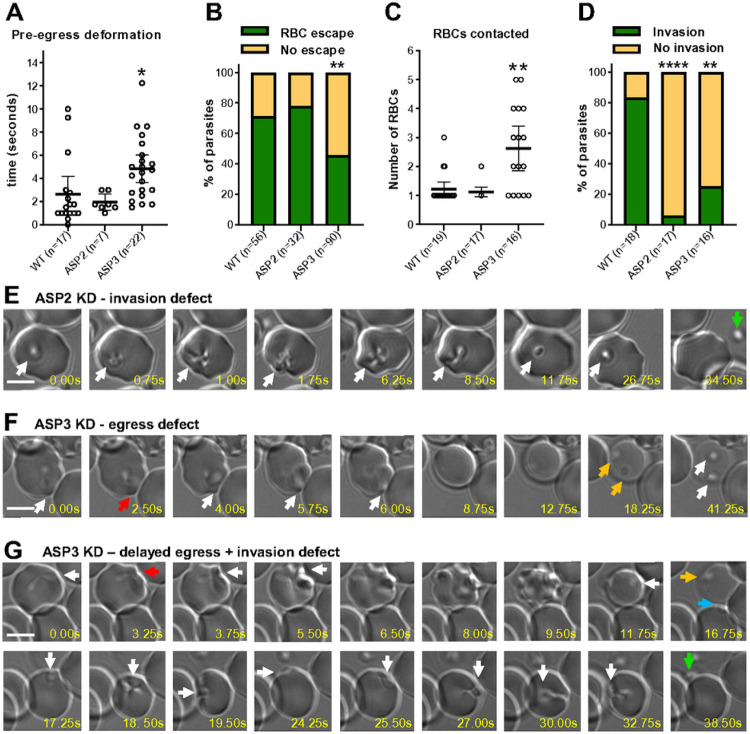
ASP2 and ASP3 are required for egress and/or invasion. **(A)** Time taken from initial deformation to RBC lysis. **(B)** Percent of parasites that escape the permeabilized RBC. **(C)** Number of RBCs contacted prior to successful invasion or becoming immotile. **(D)** Number of parasites that successful invade after contacting at least one RBC. **(E-G)** Time-lapse microscopy of 8-Br-cGMP induced egress with knockdown of ASP2 (E), ASP3 (F, G). Arrows indicate as follows: White – follows the parasites. Red – initial RBC deformation by the intracellular parasite. Orange – failed egress from a permeabilized cell. Blue – successful egress. Green – failed invasion and parasite detachment. The scale bar is 5 μm. For A and B, each dot represents an individual event. Statistical analysis in A and B is one-way ANOVA with Dunnett correction for multiple comparisons. Statistical analysis in B and D is Fishers exact test. *P<0.05. **P<0.01, ****P<0.0001.

The orthologs of *B. divergens* ASP2 and ASP3 in *P. falciparum* (PfPMIX/PMX) and *T. gondii* (ASP3) are the most upstream proteases known to date in the protease cascade that matures many proteins in the micronemes and rhoptries that are required for egress and invasion^80,81^. ASP2 and ASP3 knockdown resulted in an increased number of free merozoites in culture (Fig. 3D). In the ASP3 knockdown culture, clusters of parasites were also observed in lightly stained RBCs (Fig. 3C, ASP3 left panel). To further define these phenotypes, induced egress in knockdown parasites was observed by video microscopy. The time to egress and rate of escape from the lysed cells was the same between ASP2 knockdown and WT parasites (Fig. 4A, B and E). After egress, ASP2 parasites typically bound to a single RBC and were able to strongly deform it (pre-invasion) similar to WT, however, were unable to complete invasion and eventually detached from the cell (Figs. 4C-E; Video S5). The time from initial deformation of the RBC by the intracellular ASP3 knockdown parasite to the time of lysis of the RBC was significantly longer than in WT parasites and fewer parasites were able to escape the lysed RBC (Figs. 4A-B, F-G). Fewer ASP3 knockdown parasites were able to escape the lysed cell, and parasites that successfully egressed were able to bind to and deform the RBC, however, unlike WT or ASP2 knockdown parasites that typically bind strongly to a single host cell, ASP3 parasites maintained gliding motility over the surface of the cell, often contacting multiple host cells, but were rarely able to complete invasion (Fig. 4B-D, 4F-G; Video S6). Together, these results are consistent with the transcriptomic data suggesting ASP2 functions in the rhoptries and knockdown parasites are able to reorient but are not able to undergo the final step of invasion which requires rhoptry proteins^87-89^. The ASP3 phenotype suggests that it is required for maturation of microneme proteins that are required for RBC lysis and reorientation and/or anchoring of the apical end of the parasite to the RBC prior to rhoptry release^21,23^. In comparison to *T. gondii*, which requires only TgASP3 to process proteins in the rhoptries and micronemes, our data suggest that BdASP2 and BdASP3 are functionally orthologous to PfPMIX and PfPMX, respectively, and likely responsible for processing proteins required for invasion in the rhoptries, and egress and invasion in the micronemes, respectively.

## PKG, ASP2 and ASP3 are druggable targets required for egress and/or invasion

**Figure 5:**
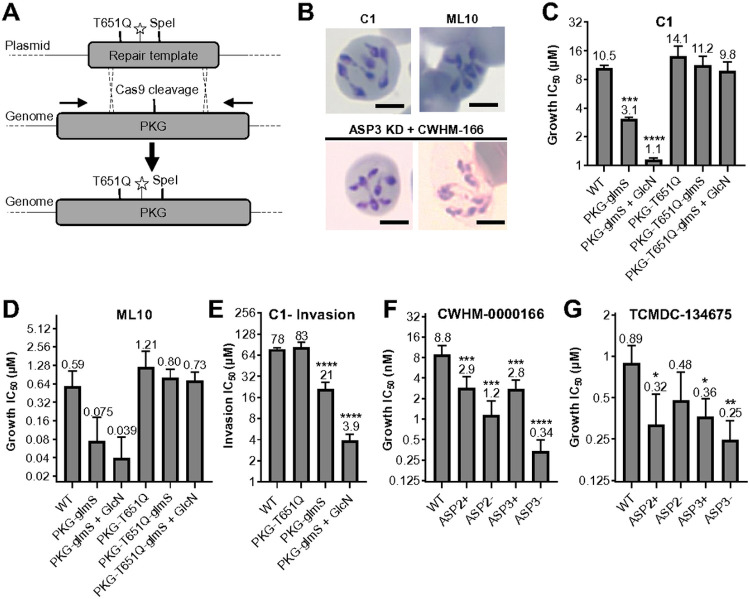
Chemical genetics reveals PKG, ASP2 and ASP3 as druggable targets in *B. divergens*. **(A)** CRISPR/Cas9 is used to introduce a putative drug resistance mutation into PKG (T651Q), a silent shield mutation (star) and a silent SpeI restriction site. **(B)** Wild-type parasites treated with 20 μM C1 or 5 μM ML10 for 48 hrs. ASP3 knockdown (No Shld1) parasites were combined with 3.4 μM CWHM-0000166 treatment for 48 hrs **(C-G)** IC_50_ of stated compound for proliferation over 3 days (C-D, F-G) or invasion (E). For C-G the data is the mean ± SD of three biological experiments performed in technical triplicate. The scale bar in B is 3 μm. Statistical analysis shown is one-way ANOVA with Dunnett correction for multiple comparisons. *P<0.05. **P<0.01, ***P<0.001, ****P<0.0001.

Significant effort has been invested to develop specific inhibitors of apicomplexan PKG, CDPKs and aspartyl proteases (PfPMIX, PfPMX and TgASP3) (reviewed in^90-94^). To demonstrate that PKG is a druggable target in *B. divergens* we used the apicomplexan PKG inhibitors compound 1 (C1), and ML10 a potent and highly specific *P. falciparum* PKG inhibitor with *in vivo* activity^95,96^. To determine the specificity of C1 and ML10 for BdPKG, CRISPR/Cas9 was used to introduce a putative resistance mutation (T651Q) equivalent to the gatekeeper mutations that confer resistance in *P. falciparum* and *T. gondii* (Fig. 5A)^67,97^. A 500 bp region within a plasmid was used as a repair template, achieving ~ 50% editing, with decreasing efficiency further from the cut site (Figure S3E and F). We utilized the ability to perform sequential transfection with the same resistance marker to generate double mutants, containing the T651Q mutation and the glmS knockdown system. Treatment of WT parasites with C1 or ML10 resulted in the same continued intracellular replication as was observed with PKG knockdown (Fig. 5B). The potency of C1 and ML10 against *B. divergens* is ~ 25-fold and 281-fold less than against *P. falciparum*, and 4.4- and 8.4-fold less than against *B. bovis*^66,96,98,99^. A 3- and 10-fold for C1, and 15- and 8-fold for ML10, reduction in the IC_50_ was observed between WT compared to PKG-glmS parasites with or without knockdown, respectively (Figs. 5C-D). A trending, but not statistically significant shift in the IC_50_ was observed between WT and the PKG-T651Q parasites (10.5 vs 14.1 μM for C1, and 591 nM vs 1209 nM for ML10), whereas the same mutation in knockdown parasites restores susceptibility to near WT levels (Figs. 5C-D). Together these results support a model in which C1 and ML10 are able to target PKG, however, at concentrations required for killing *B. divergens* there are secondary targets contributing to growth inhibition, which is likely to be CDPK4 that is the only other CDPK to share the same small gatekeeper residue as PKG (Figure S3G). The ability to rapidly and specifically inhibit PKG with C1 when combined with partial knockdown was used to investigate the role of PKG during RBC invasion. PKG knockdown increased the sensitivity to C1 invasion inhibition by 20-fold (Fig. 5E), suggesting PKG signaling is also required for host cell invasion.

To determine the druggability of ASP2 and ASP3, we screened 22 aspartyl protease inhibitors, including compounds known to be active against *P. falciparum* PfPMIX and PfPMX as well as clinical inhibitors of human beta-secretase, against WT and knockdown parasite lines (Figure S4)^81,100-103^.14 compounds displayed an IC_50_ < 25 μM, with the most potent compound (TCMDC-134675) having an IC_50_ of 891 nM against WT parasites (Fig. 5G and Table 1)^81,103^. We observe a 2.7-fold and 3.6-fold increase in sensitivity to TCMDC-134675 in ASP2 and ASP3 knockdown parasites, respectively. CWHM-0000166 was less active against WT parasites (8.8 μM), but displayed the largest increase in sensitivity of 7.7-fold and 26-fold against ASP2 and ASP3 knockdown parasites, respectively (Fig. 5F). In *P. falciparum*, incomplete PMX inhibition results in parasites lysing the PVM, whereas complete inhibition blocked PVM rupture^80,104^. To determine if the phenotype observed in ASP3 knockdown parasites was incomplete, we combined knockdown with CWHM-0000166 treatment. Under these conditions we observed that parasites continued to replicate intracellularly, as was observed with inhibition of PKG and CDPK4, further supporting a role of ASP3 in egress (Fig. 5B). We also observed clusters of parasites within a lysed RBC, in line with the live microscopy results that parasites that do lyse the host cell escape at a reduced rate. The similar phenotype seen following inhibition of ASP3 to the protein kinases CDPK4 and PKG, suggests that failure to egress by multiple mechanisms results in a default for continuation of the intracellular lytic cycle. No synergy was observed between ASP2 or ASP3 knockdown and ML10, suggesting the kinase and protease egress pathways act independently. Together these data demonstrate that PKG, ASP2 and ASP3 are essential and druggable proteins in *B. divergens* that are required for egress and/or invasion.

## Discussion

Here, we establish *B. divergens* as a genetically tractable *in vitro* model to study *Babesia* spp. cell biology. The synchronous *B. divergens* bulk transcriptome and single-cell transcriptome will serve as a resource for the study of *Babesia* spp. biology as well as for further comparative studies of apicomplexan biology. The majority of molecular research in apicomplexan parasites has been limited to *P. falciparum, P. berghei* and *T. gondii*. Transient or stable transfection systems exist for *Babesia* spp., *Theileria* spp., *Cryptosporidum* spp., *Eimeria* spp., *Sarcocystis* spp. and *Neospora* spp. (reviewed in Suarez, et al.^105^). However, the number of studies and the range of available tools and resources remains comparatively limited in these organisms. Additional studies of a wider range of apicomplexan parasites will expand our knowledge of conserved and unique biological mechanisms of apicomplexan parasitism, that are often pathogens of metazoans.

Egress is a unique aspect of apicomplexan parasitism compared to their hosts that requires specialized processes and offers novel druggable targets. The overall process of egress is similar between *Plasmodium* spp. and *T. gondii*, however, there are notable differences in their cell biology (e.g. differences in division mechanisms) and in the host cell niche that place unique pressures on the parasite (*T. gondii* resides in nucleated cells, while the asexual stages of *Babesia* spp. and *Plasmodium* spp. reside in enucleated RBCs). While the signaling and molecular mechanisms differ in some aspects, we have found that egress of *B. divergens* at the cellular level closely resembles that of *T. gondii* and has several notable differences to *Plasmodium* spp. egress despite being evolutionarily closer and sharing the same RBC niche. *B. divergens* egress begins with localized deformation of the RBC by the intracellular parasite. The deformation requires the parasites actinomyosin motor but is not necessary for disruption of the RBC membrane, although it may still contribute. Once the parasite has permeabilized the RBC membrane, it utilizes its actinomyosin motor to pass through the RBC cytoskeleton which remains relatively intact throughout egress. This is similar to *T. gondii* but in sharp contrast to *Plasmodium* spp. that fracture the RBC cytoskeleton to release parasites without requiring motility.

*T. gondii* is able to sense both intrinsic signals of parasite density and extrinsic signals of host cell damage to induce egress^22,24-27,37,106^. Similarly, *P. falciparum* can sense lipids and potassium, although their role in egress and invasion are less clearly defined^29,30^. Since *Plasmodium* spp. egress is strictly required at the end of each replication cycle, the initial egress signal is likely to be linked to the cell cycle, however, this remains to be demonstrated. The only egress signal identified in *B. divergens* is extracellular calcium, but not potassium or sodium, which acts as an extrinsic signal of host cell damage (Fig. 1E, Video S2). Whether *B. divergens* responds to its environment to determine if parasites will egress after one or two replication cycles, or whether this is regulated stochastically remains unclear.

In *T. gondii* and *P. falciparum*, the initial signals converge to either activate guanylate cyclase (GC) or inhibit PDE and thus raise cGMP levels, which in turn activates PKG. In *T. gondii*, PKAc1 suppresses cGMP levels to prevent premature egress, putatively by activating a PDE, whereas in *P. falciparum* PKAc1 is required for invasion^10,12,68-71^. Chemical inhibition or activation of PKA suggests that PKA suppresses *B. divergens* egress and is also required for invasion (Fig. 1F, S1D and S1G). It is possible these functions are performed separately by the two PKAc orthologs, although only PKAc1 displays stage specific expression (Figs. 2C). The PKAc2 knockdown line did not display an obvious growth defect and we were unable to generate a PKAc1 knockdown line. The inability to generate several of the attempted knockdown lines could be due to the DD tag interfering with the proteins function, or the proteins being sensitive to partial knockdown induced by the DD or glmS even in the absence of induction. Alternative genetic systems will need to be developed for *B. divergens* to determine the function of these proteins.

In *P. falciparum* and *T. gondii*, PKG activates PI-PLC, which generates lipid second messengers. These eventually lead to the release of calcium from intracellular stores that are required for microneme/exoneme secretion. We have demonstrated that PKG is a central component of the *B. divergens* egress signaling pathway that is both necessary and sufficient to induce egress (Figs. 1A, 1F and S3D). Small molecules targeting the lipid signaling pathway, including propranolol and U73122, had little to no effect on *B. divergens* egress and will require further genetic studies to determine the role of lipid signaling. Downstream of PKG and calcium release, CDPKs are required in *P. falciparum* and *T. gondii* egress. Of the three CDPKs expressed in *B. divergens* only CDPK4 displayed late stage expression and an egress defect when knocked down.

Inhibition of the egress signaling pathway in *B. divergens* or *B. bovis* (PKG and CDPK4 inhibition or calcium chelation) result in continued intracellular replication (Figs. 3C and E)^107,108^.

The egress signaling pathway leads to the release of the micronemes/exonemes. These organelles contain lytic factors, motility proteins and invasion ligands, many of which are proteolytically matured. In *P. falciparum*, proteolytic maturation in the rhoptries and micronemes is done in part by PMIX and PMX, respectively^81^. In contrast, *T. gondii* only contains one ortholog, TgASP3, which is localized in a post-Golgi compartment and matures a subset of microneme and rhoptry proteins^80,82^. The putative localization of BdASP2 and BdASP3 based on transcriptomics and their knockdown phenotypes suggest they are functionally orthologous to PfPMIX and PfPMX, respectively. While further work will be required to determine the substrates of these proteases and overlap between apicomplexans, we hypothesize that the continued replication phenotype observed with ASP3 inhibition is due to a block in maturation of lytic factors and therefore host cell lysis. Inhibition of either the signaling pathway or the downstream lytic factors produces a similar continued intracellular replication phenotype in *T. gondii*^40,67^. This implies that *T. gondii* and *Babesia* spp. lack a checkpoint to exit the cell cycle and that the default pathway is to continue replication. Inhibition of egress signaling in *P. falciparum* blocks replication, however, the transcriptional profile of stalled parasites continues to progress, suggesting that they may also lack a checkpoint at the transcriptional level^109^.

Small molecules have been developed that target the kinases and proteases required for egress of *Plasmodium* spp. and *T. gondii*. Identification of shared parasite targets of these compounds in *Babesia* spp. and other apicomplexan parasites will help leverage drug development efforts for malaria that have significantly more resources. Here, we have developed transfection, alongside CRISPR/Cas9 and inducible knockdown systems to modify the *B. divergens* genome. Through knockdown and the introduction of resistance mutations, we identified C1 and ML10 as inhibitors of PKG, however, at concentrations required for killing it is likely that other parasite molecules are also inhibited, with CDPK4 being the most likely target as it also contains a small gatekeeper residue (Fig. 5C-E and S3G). We also identified several compounds that dually target ASP2 and ASP3, including compounds that have known activity against PfPMIX and PfPMX lines^81,100,101^. The dual activity against these proteins may reduce the ability of the parasite to develop resistance to small molecule inhibitors. The same methods could be applied to these and other proteins to aid future drug development for *Babesia* spp. Notably, multiple new classes of potent inhibitors have been developed against PfPMX and PfPKG with drug-like properties and *in vivo* activity^104,110,111^.

Here, for the first time we have established a molecular framework for the events surrounding *Babesia* spp. egress. Several major questions remain about *Babesia* spp. egress, such as how they selectively lyse the PVM after invasion, the role of lipid signaling, the initial signal to induce egress and how they regulate egress to occur after one or more replication cycles. With the cellular, transcriptomic and genetic tools developed here, future studies will be able to answer these questions and reveal the unique biology of *Babesia* spp. as well as conserved processes throughout Apicomplexa, providing a rational basis for the development of therapeutic interventions.

## Methods

### RESOURCE AVAILABILITY

#### Lead contact

Further information and requests for resources and reagents should be directed to and will be fulfilled by the lead contact, Manoj Duraisingh (mduraisi@hsph.harvard.edu).

#### Materials availability

All unique reagents in this study are available from the lead contact. Any additional information required to reanalyze the data reported in this paper is available from the lead contact upon request.

#### Data and code availability

Single-cell RNAseq and bulk RNAseq data have been deposited at NCBI Sequence Read Archive (SRA) and are publicly available as of the date of publication. Accession numbers are listed in the key resources table. Microscopy data reported in this paper will be shared by the lead contact upon request. All code has been deposited at GitHub. DOIs are listed in the key resources table.

### EXPERIMENTAL MODEL AND SUBJECT DETAILS

#### Parasite strains

*Babesia divergens* strain Rouen 1987, kindly provided by Kirk Deitsch and Laura Kirkman (Weill Cornell Medical College), was cloned by limiting dilution (BdC9) and was maintained in purified Caucasian male O+ human RBCs (Research Blood components). The *Babesia bovis* strain MO7 was kindly provided by David Allred of the University of Florida and maintained in purified bovine RBCs (hemostat). Parasites were cultured in RPMI-1640 media supplemented with 25 mM HEPES, 11.50 mg/l hypoxanthine, 2.42 mM sodium bicarbonate, and 4.31 mg/ml AlbuMAX II (Invitrogen), at 37°C in a 1% oxygen, 5% carbon dioxide and 94% nitrogen environment.

### METHOD DETAILS

#### Induced egress

To measure egress, mixed stage parasites at 2% final HCT, 10–15% parasitemia in a total volume of 40 μL in the presence of the stated drug, were incubated at 37°C for one hour in a 96 well plate for 1 hr. Where not otherwise stated, 8-Br-cGMP was used at a final concentration of 500 μM. For inhibition of induced egress, parasites were first incubated with the stated compound at 1.33x concentration for 15 min at 37°C, prior to induced egress with the addition of 8-Br-cGMP to a final concentration of 500 μM and 1x inhibitor concentration. A final concentration of 100 μg/ml of heparin was added to prevent reinvasion. After 1 hr, the parasites were washed 3x with PBS and stained with 1:5000 Syber green II. Parasitemia was determined by flow-cytometry (Macs quant, Miltenyi) and analyzed in FloJo. Induced egress was calculated as the drop in parasitemia of the treated parasites as a percentage of the RPMI only sample.

#### Live microscopy

All videos were taken on a Zeiss Axio Observer using a 60x oil immersion lens inside a chamber heated to 37°C. Prior to imaging, parasites were allowed to settle on the bottom of a glass bottom slide (Ibidi, Cat#80827/81817) at 37°C in a 5% CO_2_ incubator for 10–15 min. Small molecule inhibitors were included in this incubation as necessary. Immediately prior to imaging, the media was removed and replaced with the RPMI/IC/EC with 500 μM 8-Br-cGMP, and any small molecules inhibitors being tested. For IC/EC experiments, the final buffer contained 0.0075% (w/v) saponin (Calbiochem, Cat#558255) to lyse the RBC. Alexa Fluor^™^ 488 Phalloidin (Invitrogen, Cat#A12379) was added to RPMI at a concentration of 1/150 where stated. All images were taken within 20 minutes of removal from the 5% CO_2_ incubator. Plasmodium falciparum mature schizonts were isolated by magnetic affinity purification (MACs LS column, Miltenyi) and imaged as per the *B. divergens* protocol. Images were processed in Zen 2 (Zeiss) and ImageJ/Fiji.

#### Isolation of free merozoites for invasion assays, synchronization and transfection

~ 1–2 ml of packed iRBCs at 20–30% parasitemia was used to isolate free merozoites using a modified protocol from ^5^. Briefly, the iRBC was resuspended to 10% HCT in RPMI and passed through two 1.2 μM filters. The isolated merozoites and RBC debris were pelleted at 3000 x g for 3 min and the supernatant was removed. For invasion assays, the merozoites were resuspended in RPMI and added to a 96 well u-bottom plate containing 1.33x final drug concentration (30 μl total) and incubated at 37°C for 10 min. Fresh RBCs were then added to a final of 2% HCT and 1x drug concentration (40 μl total). The plate was then incubated at 37°C shaking at 600 rpm for 20 min. The assay was stopped by washing the parasites three times with 200 μL PBS, 400 x g for 2 min or by adding 200 μl 4% paraformaldehyde. Parasitemia was determined by flow cytometry as per induced egress section above. For synchronization, isolated merozoites were resuspended to a final volume of 1 ml of 20% HCT RBCs in RPMI and allowed to invade for 20 min shaking at 600 rpm and 37°C. Parasites were then washed 3x with 10 ml RPMI at 400 x g to remove free merozoites and cell debris. 100 μg/ml heparin was added to prevent re-invasion throughout the time course when stated in the figure legend.

#### Plasmid construction

The sequences of all primers and synthesis products used in this study are found in table S3. The bi-directional promoter between the EF1alpha (Bdiv_030590) and LON peptidase (Bdiv_030580c) was amplified using primers BE-8 and BE-9, and cloned into XhoI/BamHI sites of pPfEF-GFP-BSD (EF1alpha side drives GFP/Cas9 expression). The DHFR (Bdiv_030660, primers BE-21/22) and HSP90 (Bdiv_037120c, primers BE-32/33) 3’UTRs were cloned into EcoRI/HindIII and SpeI/NotI sites, respectively, for the selection marker or GFP/Cas9, respectively. Cas9 was amplified from pDC2-Cas9 using primers BE-124/125 and cloned into the XhoI/SpeI sites in place of GFP. The U6 promoter, bbs1 sites (for the guide), guide tracer/scaffold, U6 terminator and PKG-T651Q repair template were synthesized by IDT (Synthesis 1) and cloned into the EcoRI site by gibson assembly to make the pEF-Cas9-PKG-T651Q plasmid. For the sgRNA, oligos were either phosphorylated, annealed and cloned into the BbsI sites, or the sgRNA was amplified using the corresponding ‘guide PCR’ primer (e.g. BE-551) and BE-550 (universal for all). For all inducible knockdown lines, the HA-glmS or HA-glmS-DD tags were amplified using primers BE-536/537. The 5’HR (HR1, 3’end of the gene) and 3’HR (HR2, 3’UTR of gene) were amplified using the corresponding HR1 and HR2 primers from Table S3 for each gene (e.g. BE_512–515 for CDPK4). The sgRNA, HR1, DD-glmS and HR2 PCR fragments were cloned into the Bbs1/PacI sites of the pEF-Cas9-PKG-T651Q plasmid in a single Gibson reaction. Correct integration of plasmids was confirmed with primers from Table S4 labelled “test integration” (e.g. BE_610/611 for CDPK4).

#### Transfection

For transfection of iRBCs using the Biorad Genepulser II, 100 μg DNA in 30 μl combined with 370 ul of cytomix (120 mM KCl, 0.15 M CaCl2, 2mM EGTA, 5mM MgCl2, 10mM K2HPO4/KH2PO4, 25mM HEPES, pH 7.6) and 200 μl iRBCs (~ 15% parasitemia). Transfection was carried out with Biorad genepulser II set to 0.31 kV, 950μF. For Amaxa nucleofection of free merozoites, merozoites were isolated from ~ 3x10^9^ iRBCs and all supernatant was removed. The pellet, containing free merozoites and cell debris, was resuspended in 100 μl of P3 solution (Lonza) plus 10 μl of water containing 2–10 μg of DNA. For Amaxa nucleofection of intact iRBCs, 10 μl iRBCs at 10% parasitemia was resuspended in 110 μl P3 + DNA solution, as per above. Transfection of either free merozoites or iRBCs was carried out in a 4D-Nucleofector System (Lonza), using the FP158 program. After electroporation of free merozoites, the parasite and buffer mixture was immediately transferred to 1 ml of RPMI containing 200 μl packed RBCs and pre-heated to 37°C. Parasites were allowed to invade at 37°C shaking at 600 rpm for 20 min before being washed with 10 ml RPMI to remove the P3 solution and returned to culture. Electroporated intact iRBCs were washed 1x with 10 ml RPMI and returned to culture. Cultures were selected with 15–20 μg of Blasticidin-S.

#### Drug sensitivity and proliferation assays

For all compounds except the aspartyl protease inhibitors, parasites were diluted to 0.2% parasitemia in 2% hematocrit (100 μl total) in the presence of the stated compounds) in a 96-well U-bottom plate. Parasites were cultured for 72 hours. Parasitemia was measured by flow-cytometry. Testing of aspartyl protease inhibitors (Fig. 5F-G, Table 1), was performed using a whole-well SYBR assay^116^ in 384-well format, with initial parasitemia set to 0.4%, 2% HCT, 40 μl/well and 72 hrs of proliferation. SYBR lysis buffer contained (0.16% saponin; 20 mM Tris-HCl; 5 mM EDTA; 1.6% Triton X 100; adjust pH to 7.4). Where not otherwise stated, +Shld was 500 nM, +GlcN was 1 mM, −Shld/−GlcN was 0 nM, except for ASP2 drug assays (Fig. 5F-G, Table 1) where + Shld was 2 mM and −Shld was 250 nM.

#### RNAseq analysis

RNA was isolated from parasites using a hybrid protocol of organic extraction combined with column purification. Briefly, parasite pellets were resuspended in 500 μi TRIzol and then extracted with chloroform. The aqueous layer was then purified using Qiagen RNeasy Mini spin-columns following manufacturers protocols. RNA was quantified and normalized into individual wells, and libraries were prepared following the Smart-seq2 protocol^117^. Libraries were sequenced on the illumina platform. **Bulk synchronous RNAseq analysis workflow:** The quality of reads was assessed using FastQC (Version 0.10.1). The reads were trimmed with Cutadapt from Trim Galore package (Version 0.3.7) (http://www.bioinformatics.babraham.ac.uk/projects/trim_galore/). The trimmed reads were mapped against the Bdivergens1802A reference genome (PiroplasmaDB release 46) and assembled with HISAT2 (Version 2.0.5 released) (https://www.ncbi.nlm.nih.gov/pmc/articles/PMC4655817/). SAM files obtained from alignment results were processed using SAMtools (Version 1.4.1) and the relative abundance of transcripts were estimated using featureCounts (https://academic.oup.com/bioinformatics/article/30/7/923/232889). **Normalization & Noise removal:** Counts per million (cpm) values per gene was calculated using cpm() from edgeR R package (Version 3.24.3) (https://www.bioconductor.org/packages/release/bioc/vignettes/edgeR/inst/doc/edgeRUsersGuide.pdf). Genes with cpm value > 2 in at least 3 samples were maintained for further analysis. Gene counts were normalized and scaled to logarithmic form using edgeR’s TMM method (trimmed mean of *M* values) with DGEList(), calcNormFactors() and cpm() functions. The cpm() parameters were as following: y = DGEList.obj, log = TRUE, prior.count = 3, normalized.lib.sizes = TRUE. Batch effected samples were Identified through the analysis of hierarchical clustering and dissimilarities method using the R function hclust() with the default parameters and logCPM expression values. These samples were excluded from further analysis. The bad samples were the ones clustered in single batches and were not similar to the rest of samples based on Euclidean distance metric. **PCA Analysis:** Principal component analysis (PCA) was run on genes with FC > 1.5 with prcomp() from R. The first 2 principal components (PCs) were chosen for visualization. **Ortholog Analysis:** Orthologous genes between *Babesia divergens* 1802A, *Plasmodium falciparum* 3d7, *Toxoplasma gondii* ME49, *Plasmodium berghei* ANKA (all from VEuPathDB release 46) were assembled using blast (version 2.10.0)(https://academic.oup.com/nar/article/36/suppl_2/W5/2505810) and the R package Orthologr (version ‘0.4.0’) with implemented best reciprocal hit method. **Smoothed expression curve analysis:** Only genes with an average of ≥ 10 cpm across each timepoint were maintained. Curves were generated using loess.smooth function in R (span = 0.5, degree = 2, family = c(‘gasussian’)). Differentially expressed genes were those that displayed a ≥ 1.5-fold change over time. *P. falciparum* data was from otto et al 2010 (downloaded from PlasmoDB). Genes with an FPKM ≥ 5 were maintained. *T. gondii* data was from Behnke et al 2010 (Downloaded from ToxoDB). *T. gondii* data was manipulated to change the timepoints to match the other datasets (i.e. it starts at 6h from paper which is straight after invasion). **Pearson correlation** was calculated in R using the smoother curves for AMA1 and RON2. Genes were considered to be highly correlated if they displayed ≥ 2-fold change over time and had a Pearson correlation value of ≥ 0.9.

#### scRNA-Seq work flow

scRNA-Seq data was processed using the 10x cell-ranger pipeline and aligned to the *Babesia divergens* 1802A genome. Counts were subsequently normalized and processed using the R Seurat package. A total of 9450 cells and 3620 genes were retained after removing cells and features with low counts (Seurat parameters: min.cells = 10, min.features = 100, nFeature_RNA > 200 nFeature_RNA < 1200). Dimensionality reduction and clustering analysis were performed using PCA and graph-based KNN as implemented in the Seurat Package. A total of 4 clusters were identified by the KNN algorithm. To make the size of the data manageable, each cluster was downsampled to include 800 cells. Global differential expression performed on each cluster (log(FC) > 1 and adjusted p-value < 0.01) identified 544 differentially expressed genes. **Pseudo-time analysis:** Pseudo-time analysis was performed on the first two PCA components by fitting a principal curve to the data and orthogonally projecting the cells on the curve. Gene expression curves were then constructed using the pseudo-time and the start of gene expression was identified by cross correlation analysis between the bulk and single cell expression curves. Further details can be found in^118^.

### QUANTIFICATION AND STATISTICAL ANALYSIS

All statistical analysis was performed in Graphpad PRISM 9. IC_50_ values were calculated using the Non-linear regression function (variable slope – four parameters, least squares regression). Statistical significance of IC_50_ changes were determined using ANOVA. All image analysis was performed in Zen 2 (Zeiss) and ImageJ/Fiji. Details of each analysis can be found in the corresponding figure legend.

## Figures and Tables

**Table 1:** IC_50_ values of Aspartyl protease inhibitors against *B. divergens*. IC_50_ (± S.D.)

	Wild-type	ASP2 (High Shld1)	ASP2 (Low Shld1 = Knockdown)	ASP3 (High Shld1)	ASP3- (Low Shld1 = knockdown)
TCMDC-134675	0.891 (± 0.307)	0.320 (± 0.212)	0.478 (± 0.288)	0.362 (± 0.129)	0.246 (± 0.095)
TCMDC-136879	4.03 (± 1.90)	1.57 (± 0.17)	1.63 (± 0.94)	1.39 (± 0.26)	0.539 (± 0.358)
CWHM-0000099	> 25	11.8 (± 0.8)	3.99 (± 3.15)	16.3 (± 3.7)	2.85 (± 1.52)
CWHM-0000117	6.81 (± 3.08)	2.66 (± 0.55)	2.56 (± 1.32)	3.17 (± 0.61)	1.13 (± 0.50)
CWHM-0000166	8.80 (± 3.18)	2.89 (± 1.27)	1.15 (± 0.70)	2.76 (± 0.96)	0.337 (± 0.157)
CWHM-0000047	19.5 (± 5.3)	18.5 (± 12.9)	6.32 (± 5.84)	16.9 (± 9.0)	8.47 (± 5.35)
CWHM-0000579	12.6 (± 6.6)	7.49 (± 4.73)	11.1 (± 6.5)	9.12 (± 6.12)	7.32 (± 6.48)
CWHM-0000162	13.3 (± 9.4)	7.15 (± 6.45)	6.47 (± 5.35)	8.00 (± 5.06)	6.41 (± 6.74)
lanabecestat	19.8 (± 5.3)	10.1 (± 3.4)	4.67 (± 3.06)	14.1 (± 2.6)	10.0 (± 5.4)
verubecestat	10.9 (± 3.3)	6.38 (± 3.27)	1.89 (± 2.78)	8.04 (± 3.36)	5.49 (± 4.29)
CWHM-0000068	16.6 (± 7.6)	15.7 (± 12.6)	8.02 (± 0.89)	13.3 (± 2.9)	9.88 (± 4.84)
CWHM-0000116	> 25	> 25	18.8 (± 8.2)	20.3 (± 11.3)	21.4 (± 10.2)
CWHM-0000123	9.46 (± 3.66)	8.39 (± 4.39)	10.8 (± 2.8)	11.2 (± 8.7)	9.22 (± 1.76)
CWHM-0000293	> 25	> 25	> 25	> 25	> 25
CWHM-0000299	> 25	> 25	> 25	> 25	> 25
CWHM-0000460	> 25	12.3 (± 10.2)	12.2 (± 1.5)	12.4 (± 5.6)	12.6 (± 4.0)
CWHM-0000580	> 25	> 25	> 25	> 25	> 25
CWHM-0000583	> 25	14.2 (± 9.5)	14.5 (± 8.9)	13.8 (± 5.1)	14.1 (± 6.9)
CWHM-0000658	16.2 (± 9.8)	12.6 (± 11.9)	6.47 (± 3.82)	9.35 (± 3.77)	7.82 (± 5.45)
AZD3839	25.7 (± 12.3)	15.8 (± 9.6)	8.64 (± 4.98)	15.8 (± 3.6)	18.6 (± 13.4)
LY2811376	24.2 (± 10.7)	6.37 (± 3.46)	2.57 (± 2.67)	13.4 (± 7.2)	12.1 (± 8.2)
LY2886721	18.5 (± 5.7)	6.38 (± 3.72)	5.76 (± 6.77)	9.62 (± 4.22)	9.30 (± 6.24)
ML10	0.864 (± 0.319)	0.794 (± 0.072)	1.56 (± 1.24)	0.682 (± 0.256)	0.628 (± 0.219)
Atovaquone	0.021 (± 0.007)	0.020 (± 0.005)	0.020 (± 0.008)	0.021 (± 0.006)	0.025 (± 0.011)

**Table:** KEY RESOURCES TABLE

REAGENT or RESOURCE	SOURCE	IDENTIFIER
**Antibodies**		
Anti-Histone H3 antibody	Abcam	ab1791; RRID:AB_302613
Anti-HA 3F10	Sigma (Roche)	12158167001; RRID:AB_390918

**Bacterial and virus strains**		
*E. coli* XL10-Gold Ultracompetent cells (all cloning)	Agilent	200315

**Chemicals, peptides, and recombinant proteins**		
8-Bromoguanosine 3',5'-cyclic monophosphate sodium salt (8-Br-cGMP)	Sigma-Aldrich	B1381
SYBR^™^ Green I Nucleic Acid Gel Stain	Invitrogen	S7563
KT5720	Sigma-Aldrich	K3761
Heparin sodium salt from porcine intestinal mucosa	Sigma-Aldrich	H3393
8-Bromoadenosine 3',5'-cyclic monophosphate sodium salt (8-Br-cAMP)	Sigma-Aldrich	B7880
**1-[6-[((17β)-3-Methoxyestra-1,3,5[10]-trien-17-yl)amino]hexyl]-1H-pyrrole-2,5-dione** (U-73122)	Sigma-Aldrich	U6756
**1,2-Bis(2-aminophenoxy)ethane-N,N,N',N'-tetraacetic acid tetrakis(acetoxymethyl ester)** (BAPTA-AM)	Sigma-Aldrich	A1076
Cytochalasin D	Sigma-Aldrich	C8273
E64	Sigma-Aldrich	E3132
Compound-1 (4-[2-(4-fluorophenyl)-5-(1-methylpiperidine-4-yl)-1H pyrrol-3-yl]-pyridine)	A gift from Dr. Jeffrey Dvorin (Boston Children’s Hospital)	
R59022 (Diacylglycerol Kinase Inhibitor I)	Sigma-Aldrich	D5919
Phenylmethylsulfonyl fluoride (PMSF)	Sigma-Aldrich	78830
*N*_α_-Tosyl-L-lysine chloromethyl ketone hydrochloride (TLCK)	Sigma-Aldrich	T7254
N-p-Tosyl-L-phenylalanine chloromethyl ketone (TPCK)	Sigma-Aldrich	P5318
Pepstatin A	Sigma-Aldrich	P5318
Chymostatin	Sigma-Aldrich	C7268
E64d	Selleckchem	S7393
ALLN (Calpain inhibitor 1)	Sigma-Aldrich (Calbiochem)	208719
H89	Sigma-Aldrich	B1427
BIPPO 5-benzyl-3-isopropyl-1H-pyrazolo[4,3-d]pyrimidin-7(6H)-one	A gift from Dr. Jeffrey Dvorin (Boston Children’s Hospital).	
A23187	Sigma-Aldrich	C7522
Propranolol hydrochloride	Sigma-Aldrich	P0884
ML10	A gift from Dr. Simon Osborne (LifeArc)	
Saponin	Calbiochem	558255
Alexa Fluor^™^ 488 Phalloidin	Invitrogen	A12379
Shld1	Synthesized as per(Banaszynski et al., 2006)	
TRIzol^®^	Invitrogen	15596026
D-(+)-Glucosamine hydrochloride (GlcN)	Sigma-Aldrich	G1514
Blasticidin-S	Invivogen	ant-bl-10p
**Critical commercial assays**		
DNeasy Blood and Tissue Kit	Qiagen	69504
RNeasy Mini spin-columns	Qiagen	74106
P3 Primary Cell 4D-Nucleofector^™^ Kit	Lonza	V4XP-3024

**Deposited data**		
Bulk synchronous RNAseq	NCBI SRA	PRJNA804502
Single-cell RNAseq	NCBI SRA	PRJNA803312
Code	GitHub	https://github.com/umbibio/Babesia_time_course


**Experimental models: Cell lines**		
*Babesia divergens* strain Rouen 1987	Kindly provided by Kirk Deitsch and Laura Kirkman (Weill Cornell Medical College)	

**Oligonucleotides**		
See supplementary table 3 for primers and synthesis products		

**Recombinant DNA**		
pBdEF1-GFP-BSD	This study	
pBdEF1-Cas9-BSD-PKG-T651Q	This study	
pBdEF1-Cas9-BSD-CDPK4-HA-DDglmS	This study	
pBdEF1-Cas9-BSD-CDPK5-HA-DDglmS	This study	
pBdEF1-Cas9-BSD-CDPK7-HA-DD-glmS	This study	
pBdEF1-Cas9-BSD-PKAc1-HA-DD-glmS	This study	
pBdEF1-Cas9-BSD-PKAc2-HA-DD-glmS	This study	
pBdEF1-Cas9-BSD-ASP2-HA-DD-glmS	This study	
pBdEF1-Cas9-BSD-ASP3-HA-DD-glmS	This study	
pBdEF1-Cas9-BSD-DPAP1-HA-DD-glmS	This study	
pBdEF1-Cas9-BSD-PLP1-HA-DD-glmS	This study	
pBdEF1-Cas9-BSD-PLP3-HA-DD-glmS	This study	

**Software and algorithms**		
Prism version 9	GraphPad	
ImageJ/Fiji version 2.0	https://imagej.net/software/fiji/	
Zen 2	Zeiss	
VEuPathDB release 46 (PiroplasmaDB/PlasmoDB/ToxoDB)	https://veupathdb.org/veupathdb/app	

**Other**		
8/18-well glass bottom imaging chamber	Ibidi	80827/81817
1.2 μM Pall Acrodisc^®^ Sterile Syringe Filters with Supor^®^ Membrane	Pall	4656
